# Comparison of classic Sanger and next generation sequencing mitotypes of second world war victims from Konfin I mass grave

**DOI:** 10.1007/s00414-025-03603-1

**Published:** 2025-09-15

**Authors:** Marcel Obal, Irena Zupanič Pajnič

**Affiliations:** Institute of Forensic Medicine, Faculty of Medicine, University of Ljubljana, Korytkova 2, Ljubljana, 1000 Slovenia

**Keywords:** MtDNA, Next generation sequencing, Sanger sequencing, WWII, Skeletal remains

## Abstract

Rapid technological advancements have significantly enhanced DNA analysis. A key innovation is Next-Generation Sequencing (NGS), also known as Massively Parallel Sequencing (MPS), which followed classic Sanger (CS) sequencing. Compared to CS, NGS offers higher sensitivity, resolution, and throughput, making it particularly valuable for mitochondrial DNA (mtDNA) analysis. The high copy number, matrilineal inheritance, and non-recombining nature of mtDNA, especially its hypervariable regions (HV), make it highly relevant in forensic investigations. NGS has introduced streamlined protocols and improved low-level heteroplasmy detection in mtDNA sequencing. However, with any new technology, its informativeness and authenticity must be evaluated against traditional methods. This study compared mitotypes from degraded WWII skeletal remains recovered from a Slovenian mass grave, using the same DNA extraction method to minimize pre-sequencing variability. Femurs were mechanically and chemically cleaned, pulverized, and fully demineralized. DNA was extracted and purified using EZ1 Advanced XL and quantified with an in-house protocol. CS sequencing was performed using BigDye Terminator Kit v1.1 and ABI PRISM™ 3130 Genetic Analyzer, while NGS was conducted with the Precision ID mtDNA Control Region Panel and Ion GeneStudio™ S5 System. Comparison of mitotypes revealed that NGS identified low-level heteroplasmies undetectable by CS, particularly in length heteroplasmy. However, since Ion Torrent™ Suite 5.10.1 is prone to errors, certain NGS variants had to be disregarded.

**Supplementary Information:**

The online version contains supplementary material available at 10.1007/s00414-025-03603-1.

## Introduction

The lack of recombination, high copy number (CN) per cell, circular shape, and inheritance through the maternal line are the main reasons that mitochondrial DNA (mtDNA) is significantly appealing when it comes to forensic genetic analyses. This is most evident when only samples that are rather challenging to analyze are available, as mtDNA is more resistant and abundant than nuclear DNA [1–4]. Segments of mtDNA referred to as hypervariable (HV) regions, are known for their significant genetic diversity and thus are shown to be favored in forensics, for they allow good differentiation of individuals, and the genetics populations they belong to [5, 6].

The last decade has seen a rapid progress in the development of novel techniques. Succedent to the Classic Sanger (CS), use of next generation sequencing (NGS) technologies has been on the rise [7]. With possibilities such as the capacity to simultaneously analyze a large number of markers with a significantly higher throughput, higher resolution, and higher sensitivity [8–12], NGS offers a plethora of advantages in forensic analyses when compared to CS sequencing. The latter also reflects in mtDNA analyses. Originally perfomed with CS sequencing and despite improved protocols being developed [13], mitotype analysis tends to be less laborious, faster, and offers improved low-level heteroplasmy detection when performed with NGS [14], and even enables improved mixture interpretation [11, 15]. It has previously been shown that heteroplasmy pattern and level can vary between tissues, which should be addressed when analyzing mitotypes [16].

Moreover, when new technologies are introduced an essential first step is a comparison to the older ones. In this manner informativeness and credibility of the results are assessed and established. This task should be undertaken by each laboratory that is planning to introduce new techniques into laboratory workflow, with an emphasis on considerations that best tailor to laboratory’s needs. For instance considering what types of samples are used and their quality, and which types of analyses are performed. Studies have suggested that variations in outcomes are possible, such example being mitotype mismatches between CS sequencing and NGS that have been observed when using different tissues as samples (buccal swabs and hair) for comparing low-level heteroplasmy [16]. Whereas various studies have successfully pursued the task of comparing the two aforementioned technologies [17–20], and some also using different tissue samples [21, 22], none have joined the following: mtDNA HV regions I and II analysis, isolated from lesser quality old bones while using the same extraction method - in order to ensure that pre-sequencing experimental conditions are as comparable as possible.

This study aims to compare mitotypes acquired by using two different methods – CS sequencing and NGS Ion Torrent (Thermo Fisher Scientific, Waltham, United States) – in order to see if the results are concordant, and to find and identify any missing or additional information that can be used and which affects the identification process in forensic analyses.

## Materials and methods

### Sampling

Parts of right femurs belonging to 17 victims found in a WWII mass grave Konfin I were sampled. The grave is located in Slovenia and has been described in previously published research. With the exception of sample S2, other samples have previously been identified using autosomal as well as mtDNA, and, where applicable, Y-chromosome markers, by matching them to the living relatives [23], which was used as sequence authenticity criteria. We found poorly preserved skeletal remains to be a good representative model of relevant forensic samples, as we often encounter skeletal remains of missing persons in our routine casework.

### Contamination prevention

Well established protocols for contamination safeguard as well as for sample preparation were followed. The cleaning protocol for the instruments that were used included cleaning with 6% sodium hypochlorite, bidistilled water rinsing (Millipore, Darmstadt, Germany), 80% ethanol (Fisher Scientific, Loughborough, UK) rinsing, and sterilization. Directly prior to use all of the plastics, reagents, and tools used were placed under UV light. Disposable nitrile gloves, as well as disposable caps and gowns were used by the persons handling the samples, and STR profiles based elimination database was created.

### Sample Preparation

A specially designed room for working with old bones was used for sample preparation, which included eliminating the outer layer to remove any present contamination agents on the surface. A rotary tool (Schick, Schemmerhofen, Germany) was used, and the process carried out in a fume hood (Iskra Pio, Šentjernej, Slovenia). Chemical cleaning involving a 5% Alconox detergent (Sigma-Aldrich, St. Louis, MO, USA), bidistilled water wash, and final wash with 80% ethanol followed. After being airdried overnight, the femur pieces were broken into smaller pieces, and then ground to dust using a homogenizer - Bead Beater MillMix 20 (Tehtnica, Domel, Železniki, Slovenia) – at 30 Hz for 1 min. The grinding jars cooled with liquid nitrogen in order to prevent DNA damage. To accomplish full demineralization, 0.5 g of powdered bone was incubated in 10 ml of ethylenediaminetetraacetic acid (EDTA; Promega, Madison, WI, USA) overnight. The details of the protocol followed can be found in previously published literature [24].

### DNA extraction and purification

Automated extraction and purification of DNA was conducted using EZ1 Advanced XL (Qiagen, Hilden, Germany) instrument alongside the EZ1 DNA Investigator Kit (Qiagen), with trace protocol chosen, and 50 µl of DNA in a Tris EDTA buffer solution elution. Protocols provided by the manufacturer were followed, and extraction negative controls (ENCs) were analyzed in parallel to oversee potential contamination.

### MtDNA quantification

A previously described and published in-house quantitative PCR (qPCR) method for determining mtDNA copy number (CN) was used for quantification [25]. It determines copy number based on 113 bp long fragment enclosed by two custom primers by Applied Biosystems (AB; Renfrewshire, UK), and uses a 620 bp fragment as a standard. Though based on the study by Alonso [26], we used TaqMan™ Universal Master Mix II with Uracil-N-glycosylase (TFS), as the one used in the original study is not available anymore. All samples were measured in duplicates using QuantStudio™ 5 Real-Time PCR System (TFS), and Design and Analysis Software v1.5.2 (TFS) was used for subsequent raw data analysis.

### Sequencing and data analysis

#### Classic Sanger sequencing

PCR protocol and primer sequences are described in formerly published study by Parson et al. [27]. PCR, carried out in Biometra UNO-Thermoblock (Biometra, Göttingen, Germany), was followed by ultrafiltration purification using Centricon-100 (Millipore Corporation) filter units. BigDye Terminator Cycle Sequencing Ready Reaction Kit, version 1.1 (AB) and ABI PRISM™ 3130 Genetic Analyzer (AB) were used for sequencing. Data was recorded with Data Collection v 3.0 in AB DNA Sequencing Analysis Software v 5.2 (AB) and compared to a reference sequence using BioEdit (http://en.bio-soft.net/format/BioEdit.html).

#### Next generation sequencing

For automated library preparation the HID Ion Chef™ Instrument (TFS) was used alongside the following two kits: Precision ID DL8 Kit™ (TFS) and Precision ID mtDNA Control Region Panel (TFS). The entire process was carried out in line with manufacturer’s recommendations [28] with the the following settings: 22 cycles, 2 primer pools, 4 min of anneal and extension time. Each library was quantified on a QuantStudio™ 5 Real-Time PCR System (TFS) using Ion Library TaqMan™ Quantitation Kit (TFS), and data analyzed with Design and Analysis Software v1.5.2 (TFS). Templating onto Ion 530™ Chip (TFS) was performed using Ion Chef™ Instrument (TFS) and Ion S5™ Precision ID Chef Supplies, Ion S5™ Precision ID Chef Solutions (TFS), and Ion S5™ Precision ID Chef Reagents, following the official protocol [28]. Sequencing was performed using Ion GeneStudio™ S5 System (TFS), Ion S5™ Precision ID Sequencing Reagents and Ion S5™ Precision ID Sequencing Solutions (TFS). Raw data primary analysis with sequence alignment to the revised Cambridge Reference Sequence and variant calling was performed using Ion Torrent™ Suite 5.10.1 (TFS) with HID Genotyper 2.2 and Coverage Analysis (v5.10.0.3) plugins using default settings. Minimal total read coverage for a variant call, and minimal variant coverage to be considered for variant calling were both set at 20. Threshold percentage for confirmed call was set at 96. Threshold for a point heteroplasmy call was set at 10, threshold for an insertion call was set at 20, and threshold for a deletion call was set at 30. Subsequent secondary analysis was performed using Converge™ Software v2.3 (TFS) in order to visualize data, and default analysis settings with the same thresholds as those set in Ion Torrent™ Suite 5.10.1 (TFS) were applied. Mitotypes generated with the Ion Torrent™ Suite 5.10.1 (TFS) with HID Genotyper 2.2 were utilized in the comparison to CS. Additionally, sequence quality control (QC) for each sample was performed using CLC Genomics Workbench 24 (QIAGEN) through visualizing quality distribution, GC-content, and lengths distribution. Default settings with no hard cuttoffs were used. High quality sequences are considered those that have quality distribution with an average PHRED score of 30 for majority of sequences in a sample, with a normal GC-content distribution, and had no unexpected length distribution peaks.

## Results

Supplementary Material (SM) 1 shows quantification results in copies of mitogenome per µl. Quantities ranged from 11,595 (S14) to 554,458 (S2) copies/µl. Concentrations were normalized to 5000 copies/µl before sequencing.

SM 2, Table 1 contains NGS QC and sequencing data for each sample. For Ion Torrent™ Suite 5.10.1 analysis mapped reads, on Target reads, mean depth, and uniformity are shown for each sample. Mapped reads ranged from 136,510 (S1) to 361,408 (S12), on Target reads were between 99.84% (S1 and S14) and 99.98% (S6 and S7), mean depth ranged from 11,789 to 32,686, and uniformity was between 70.64% and 91.48%.

There were 740 M total bases, and 6,202,091 total reads, out of which 19% were usable. Ion Sphere Particle (ISP) loading was 85%. Mean read length was 119 bp. There were 660 M total alignment bases, 92.2% of reads were aligned, and 39620.3X was the average coverage depth of reference.

SM 2, Table 1 portrays graphical report of CLC Genomics Workbench 24 (QIAGEN) analysis - quality distribution, GC-content, and lengths distribution are shown for each sample. The majority of sequences in all of the samples had average PHRED score of 30 (scores 20 and above are considered good quality), GC-content proportions between 40 and 60% (indicating mostly human DNA, as percentages above and below this range can indicate the presence of contaminants), and lengths distribution in concordance with the sequencing method between 100 and 200 bp). However, there are multiple peaks present in GC-content distribution, which could indicate contamination with non-human DNA.

SM 33 displays side by side comparison of mitotypes resulting from two different methods for each respective sample. Mitotypes obtained with CS sequencing (as well as autosomal STR profiles) were previously matched to those from victims’ relatives (data not shown).

HVI and HVII regions were sequenced with CS, therefore only those two regions were included in comparison, even though the whole control region was sequenced with NGS. The two compared mtDNA regions are restricted to the nucleotide positions 16,030–16,400 (HVI region) and 55–407 (HVII region). The non-included parts of control region were therefore HVIII region, and the small inter-regional portion between HVI and HVII (where variants such as 16519 C could be observed).

76% of samples matched between all compared variants, 12% of samples had undetected missing variants only when analyzed with CS sequencing, 12% had undetected variants only when analyzed with NGS. Variants that were not detected using either CS sequencing or NGS are crossed out. Differences in single nucleotide polymorphisms (SNPs), and heteroplasmic major variants were rarely observed and were concordant between CS sequencing and NGS. In general the differences were observable in the form of detected variants only when NGS was used, compared to CS sequencing where the same variants were not detected. Predominantely the variants that were observed only in NGS were heteroplasmies. However, minor variant length heteroplasmies (deletions), such as 309del, and 249del were disregarded from comparison in cases where the variant was only detected by NGS, for the presence of these variants might be a direct result of the software used. For respective samples these variants are shown in the Disregarded variants column. Variants that are colored blue are point heteroplasmies and were detected as such only using NGS. Samples S3, and S12 had variants that were reported in CS analysis, but were not reported in NGS analysis. For sample S3 that includes variants 263G and 295 T, and for sample S12 that is variant 309.2 C.

In two samples variants were observed in CS but not in NGS, which tends to be rather unusual as NGS has higher sensitivity. This could therefore be attributed to a possible drop out event or a potential sequence misalignment. In both cases (variants 263G and 295 T in S3) there was no coverage at all at these positions, therefore the thresholds set described earlier could not be met, and the variants could not be detected.

## Discussion

QC analysis results of the NGS data indicated a high base call accuracy with PHRED scores, possible artifacts through multiple peaks observed in GC-content distribution, and expected lengths distribution.

The results reflected both the advantages as well as disadvantages of the NGS platform (Ion GeneStudio™ S5 System (TFS)) that was used. The most notable example is that low-level heteroplasmies were being observed only in NGS, which coincides with the fact that NGS is in general much more sensitive and thus has lower threshold of detection [12]. Greater sensitivity of NGS can be advantageous (especially in low-quality and low-quantity DNA samples) because the variants that were previously undetectable with CS can now be detected with NGS and help towards a higher discrimination power and better resolution in human identification. However, detection of low-level variants with NGS also introduces certain considerations such as incomparability with forensic CS-based databases and sequence authenticity, for the variants in question could be a result of contamination or sequencing error. This further emphasizes that comparison between CS sequencing and NGS should be done not only when different, less challenging samples (such as buccal swabs or hair) are at play [16], but also when an extract from the more challenging to analyze tissue, such as aged bones, is used, because differences in heteroplasmic variants can occur [29]. Moreover, it is important to establish heteroplasmy interpretation parameters for challenging samples for differentiation between heteroplasmy and damage might be needed in the analysis [30]. Furthermore, the inconsistencies that were observed in length heteroplasmies, where certain poly-stretches did not match in length, are most probably a result of limitations of the IonTorrent™ technology. In such cases where there are length variations in a sequence, the technology might not be able to precisely differentiate the signal to determine how many of the same bases are there, thus potentially compromising the sequencing resolution [31]. When analyzing Ion Torrent sequencing data, especially in homopolymeric regions it’s crucial to pay attention to potential sequencing artifacts, such as insertion and deletion errors, subsequent indel miscalls because of misalignments, strand bias artifacts, and coverage dropouts [32]. In line with studies that suggest excluding certain variants, namely length heteroplasmies, as they might not be reliable when comparing mitotypes for forensic purposes, we chose to take the same approach. However, opting for length heteroplasmy exclusion in order to be able to compare two mitotypes analyzed with CS and NGS respectively could impact accuracy and discrimination potential [16], and differentiation between maternal relatives [29]. However, it has been suggested by Šenovská et al. [33] that when working with ancient DNA (aDNA) CS might be used as a pre-NGS screening, helping to identify potential contamination as well as assess the suitability of the samples for further NGS analyses, thus optimizing the costs.

The aimed comparison of mitotypes was overall successful, as well as additional information (such as low level variants) was obtained with the more sensitive NGS method, which is particularly appreciated when working with such challenging samples. However, the use of this additional information obtained might have to be advised against in identification processes, because the sequencing method exhibits certain shortcomings that could be affecting the results.

## Conclusion

Through using old-bone-derived mtDNA for mitotype comparison between CS sequencing and NGS technologies, it was possible to re-emphasize the importance of establishing credibility and informativeness of the results, based on the type of tissue as well as the type of analyses that are carried out in our routine casework. Mitotype comparisons should therefore be done using the same extraction methods, and sequencing technologies, in order to be the most informative and discriminative.

## Supplementary Information

Below is the link to the electronic supplementary material.

Supplementary Material 1 (XLSX 10.5 KB)

Supplementary Material 2 (XLSX 4.01 MB)

Supplementary Material 3 (XLSX 17.1 KB)

## Data Availability

The authors declare that all the data are available upon request.
